# Flow cytometric analysis of CK19 expression in the peripheral blood of breast carcinoma patients: relevance for circulating tumor cell detection

**DOI:** 10.1186/1756-9966-28-57

**Published:** 2009-04-28

**Authors:** Lili Wang, Yanyan Wang, Yajing Liu, Min Cheng, Xu Wu, Haiming Wei

**Affiliations:** 1 Hefei National Laboratory for Physical Sciences at Microscale and School of Life Sciences, University of Science and Technology of China, Hefei, Anhui 230027, PR China

## Abstract

**Background:**

Immunocytochemistry and RT-PCR have been widely used for the detection of circulating tumor cells in patients with breast cancer but their specificity is limited. Our purpose is to utilize a convenient and specific technology to detect circulating tumor cells in breast cancer patients.

**Methods:**

To determine the sensitivity and specificity of our method, A431 cells were serially diluted with human peripheral blood leukocytes and stained with CK19. A total of 73 blood specimens including 25 healthy volunteers and 48 patients with breast carcinoma and benign tumor were tested by flow cytometry to quantify the expression of CK19.

**Results:**

The detectable upper limit of A431 cells was 1 cancer cell among 10^4 ^human white blood cells. CK19 was detected in 27% of breast cancer patients but none control gives positive result. The number of cancer cells increased gradually along with the disease stages for it was the least in stage I (0%) and the most in stage IV (1.29%). Fifteen patients were observed during three month chemotherapy after surgery, and most of their CK19 expression levels declined after treatment.

**Conclusion:**

Our research convinces that the detection of CK19 in peripheral blood by flow cytometry is also a specific and feasible method to monitor circulating tumor cells in breast cancer.

## Background

Nowadays breast cancer is becoming the second leading cause of cancer deaths in females, almost 10% women have the risk of developing breast cancer [1]. Although great improvements have been made in curing breast cancer, the overall five-year survival rate remains < 50% and many patients relapse after surgical resection because of the dispersion of undetectable cancer cells [2,3]. Therefore, it is necessary to establish sensitive and specific techniques for the detection of occult tumor cells. A better method for early diagnosis may help in predicting recurrence and planning appropriate therapies to improve survival [4,5].

Many investigations have indicated that epithelial cells from the initial tumor can be recognized in peripheral blood or bone marrow aspirates of patients with breast cancer [6,7]. The detection of circulating tumor cells (CTCs) in the peripheral blood of cancer patients has been associated with recurrence and metastasis of breast cancer [8-10]. Cytokeratins (CKs), characteristic intermediate filament of epithelial cells, especially CK19, are widely used to detect tumor cells derived from epithelial tissues [11,12]. CK19 has been shown to correlate with poor clinical outcome for patients with breast cancer [13].

Flow cytometry is a technology which can not only give information of high statistical precision and subpopulation quantification but also analyze cells individually and rapidly, compared with immunocytochemistry [14,15] and reverse transcriptase polymerase chain reaction (RT-PCR) [16,17]. In this study, flow cytometry was used to detect occult tumor cells in peripheral blood of patients with breast cancer. The detection of CTCs in peripheral blood of 48 patients was intended to find the relationship of CK19^+ ^cell percentage with disease progress. CK19 was positive in the peripheral white blood cells of breast cancer patients at stages II to IV, but not the patients at stage I and healthy controls. The percentage of CK19^+ ^cells was increased following the severity of the disease and decreased after lumpectomy and chemotherapy.

## Methods

### Cell line

The A431 (human epithelial carcinoma) cell line obtained from the American Type Culture Collection was grown in Dulbecco's modified Eagle's medium (DMEM) supplemented with 15% fetal calf serum (both from GIBCO), 100 U/ml penicillin, and 100 μg/ml streptomycin at 37°C in a humidified incubator with 5% CO_2_. Subculture was performed when confluence reached 70%.

### Patients

Breast cancer patients were treated at the Affiliated Hospital of Anhui Medical University. The cohort included 7 patients with benign tumor, 34 patients with primary breast cancer and 7 patients with metastatic breast cancer from October 2006 to April 2008. The patients underwent lumpectomy except those with distant metastases. And we detected CK19 expression of 15 patients with primary breast cancer during three month chemotherapy. Blood samples were obtained with informed consent after approval of the protocol by the Ethics Committee of the University of Science and Technology of China. Control blood samples were collected from 25 healthy female volunteers.

### Blood sample preparation

The first 8 ml of blood was discarded to avoid epithelial contamination before the collection of 5 ml blood sample. Human white blood cells were isolated from adult peripheral blood using RBC lysis buffer (RX-2-1-2 U-gene China). Briefly, 3 ml blood and 15 ml RBC lysis buffer were mixed with vortex and kept on ice for 15 min until pellucid, then were centrifuged at 450 g for 10 min. Cells were suspended with 5 ml RBC lysis buffer and centrifuged at 450 g for 10 min again followed by twice rinse with PBS.

### Immunofluorescence staining

A431 cells were counted onto glass slides at a concentration of 5 × 10^5 ^cells per spot. Subsequently, the cells were fixed with 4% paraformaldehyde in PBS for 15 min at room temperature, rinsed in PBS, and incubated with FITC-conjugated mouse anti-human CK19. Laser scanning confocal microscopy was performed and the data were processed with MetaMorph program.

### Flow cytometric analysis

After fixation with 1% paraformaldehyde for 1 hour at room temperature, A431 cells or leukocytes were permeabilized with 0.01% Triton X-100 for 1 h at room temperature followed by mouse serum block (30 min, room temperature). Then the cells were incubated with FITC-conjugated CK19 antibody or FITC-mouse IgG_1 _isotype antibody (both from BD PharMingen) as negative control overnight. After washed twice with permeabilization buffer, samples were analyzed by FACSCalibur (Becton Dickinson).

### Statistical analysis

K Related Samples Test was used for the analysis of CK19 expression in peripheral blood of patients before and after clinical treatment. Mann-Whitney U test was used to compare CK19 expression levels in peripheral blood between patients at stage III and stage IV. The statistical significance was defined as values of p < 0.05.

## Results

### CK19 expression in A431 cells

**Figure 1:**
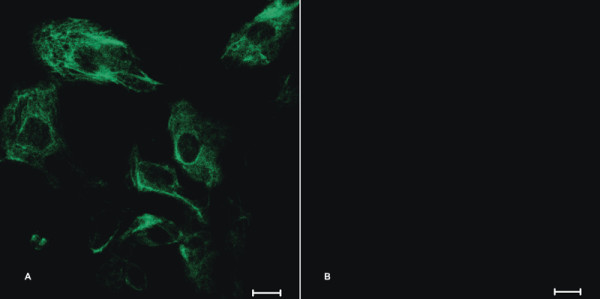
**Detection of CK19 expression in A431 cells by immunofluoresence staining**. A431 cells were incubated with FITC-conjugated CK-19 antibody (A) or FITC-mouse IgG_1 _(isotype control) (B) and analyzed the expression of CK19. The scale bar = 20 μm.

Immunofluorescence staining was used to detect the CK19 expression in A431 cells. The result showed that A431 cells were CK19-immunoreactive cells and CK19 was mainly located in the cytoplasm of A431 cells (Figure 1).

### The specificity and sensitivity of flow cytometry

**Figure 2:**
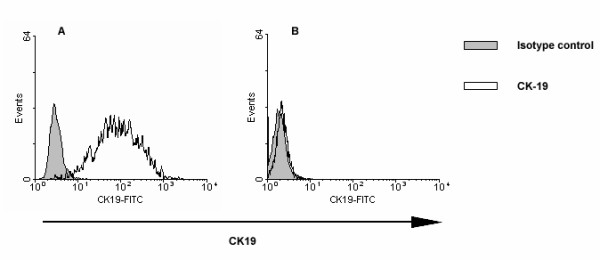
**CK19 expressions in A431 cells (A) and human white blood cells (B)**. The cells were fixed, permeabilized with 0.01% Triton X-100, stained with FITC-conjugated mouse anti-human CK19 antibody or FITC-conjugated mouse IgG_1 _and analyzed by flow cytometry.

**Figure 3:**
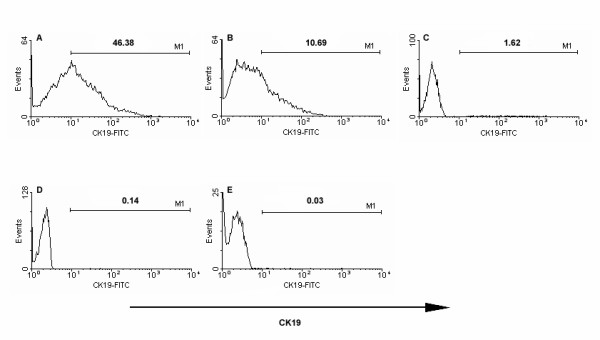
**Expression of CK19 in A431 cells diluted with human white blood cells at different ratios**. A431 cells were mixed with healthy adult white blood cells at different ratios of 1:1 (A), 1:10 (B), 1:10^2 ^(C), 1:10^3 ^(D), and 1:10^4 ^(E). The cell mixture was stained with FITC-anti-CK19 antibody and detected the expression of CK19.

Intracellular flow cytometric analysis indicated that all the A431 cells expressed high level of CK19 (Figure 2A). However, healthy adult peripheral blood white blood cells had no CK19 expression (Figure 2B) (n = 25). A431 cells were mixed with healthy adult white blood cells at different ratios of 1:1, 1:10, 1:10^2^, 1:10^3^, and 1:10^4 ^to determine the sensitivity of flow cytometry. It showed that the percentages of CK19^+ ^cells detected by flow cytometry were consistent with the ratios of A431/white blood cells. Flow cytometry could distinguish the very low percentage of CK19 expressing cells, even 1 A431 cell in 10^4 ^white blood cells. It suggested that flow cytometry had specificity and sensitivity to examine CK19 expression and possessed the potential to detect the few circulating breast cancer cells in the whole blood samples (Figure 3).

### Patient characteristics

The characteristics of 48 patients enrolled in the study are listed in Table 1. The age range of patients was from 28 to 82 years old and the median age was 46 years old. There were 41 patients with no evidence of metastasis in the liver and bone and 7 patients with metastatic breast cancer. All the patients with breast cancer were clinically classified as stages I to IV. The patients with primary breast cancer were performed lumpectomy followed by chemotherapy. Thirteen of the 48 patients (27%) were found to have CK19^+ ^cells in peripheral blood including 7 patients with primary breast cancer and 6 with metastatic breast cancer (Table 2).

**Table 1:** Details of patients and CK19 expression in peripheral blood

	Number of patients	%	Positive cases
Pathology size			

< 1 cm	5	10.4	1

1–2 cm	11	22.9	3

> 2 cm	32	66.7	9

Clinical stage			

Benign tumor	7	14.6	0

I	4	8.3	0

II	23	47.9	2

III	7	14.6	5

IV	7	14.6	6

Histology			

Infiltrating ductal carcinoma	39	81.3	13

Fibroadenoma	1	2	0

Struma	6	12.5	0

Intraductal breast cancer	2	4.2	0

Distant metastasis			

Metastasis	7	14.6	6

Without metastasis	41	85.4	7

Note: The patient age ranged from 28 to 82 years old.

**Table 2:** Overview of CK19^+ ^results in volunteers, benign tumor patients and stage I–IV breast cancer patients

	Total number	Positive Detection Rate
Healthy control	25	0/25 (0%)

Benign tumor	7	0/7 (0%)

Stage I patients	4	0/4 (0%)

Stage II patients	23	2/23 (9%)

Stage III patients	7	5/7 (70%)

Stage IV patients	7	6/7 (86%)

### Detection of circulating breast cancer cells in peripheral blood of patients before surgery by flow cytometry

**Figure 4:**
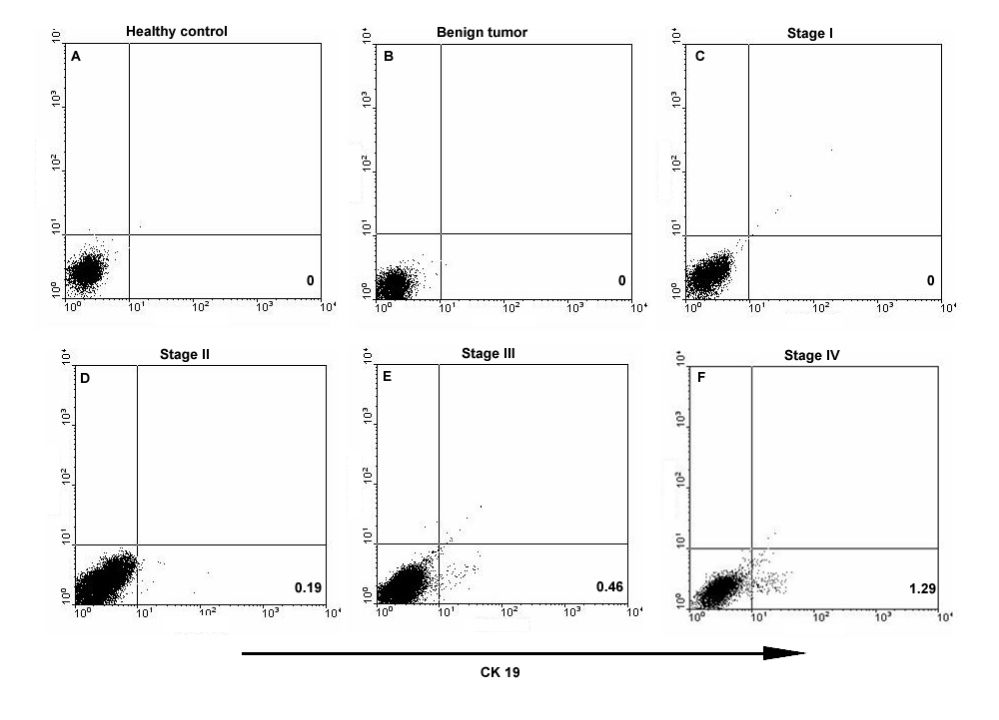
**CK19 expression in peripheral blood of healthy controls and breast tumor patients**. Peripheral white blood cells were isolated and stained with FITC-conjugated mouse anti-human CK19 antibody to examine CK19 expression. (A) Healthy volunteers; (B) Benign tumor patients; Breast cancer patients at stage I (C), stage II (D), stage III (E) and stage IV (F).

**Figure 5:**
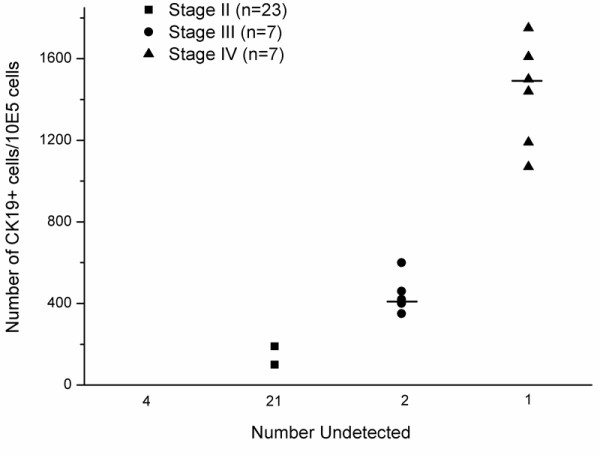
**The expression level of CK19 in peripheral blood of breast cancer patients is correlated with the disease stage**. CK19 from each peripheral blood sample was detected by flow cytometry as described in methods. All the negative results were shown as number undetected. All 4 patients at stage I were CK19 negative. The median is marked as "---" in each group. Where the frequency of negative cases is > 50%, the median cannot be shown. p < 0.05 was considered significant.

Flow cytometric analyses showed that no CK19 was expressed in peripheral blood of healthy control (n = 25), benign tumor patients (n = 7) and breast cancer patients at stage I (n = 4) (Figures 4A, B, C). But there existed CK19^+ ^cells in the peripheral blood samples of patients at stages II, III, and IV (Figure 4D, E, F), with the median of each group of 0.15% (n = 2), 0.44% (n = 5) and 1.47% (n = 6) (Figure 5), respectively. There was significant difference in CK19 expression between patients at stage III and stage IV (p = 0.0043).

### The change of CK19 expression in 15 patients with breast cancer during 3 month-chemotherapy

**Figure 6:**
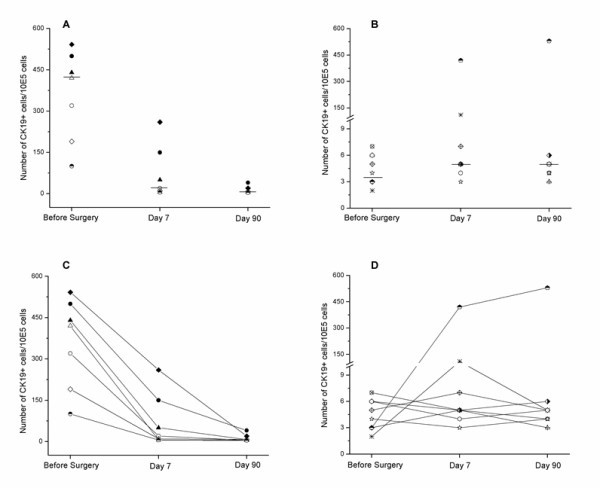
**The CK19^+ ^cell number in peripheral blood of 15 patients with primary cancer before surgery and after chemotherapy**. All the patients underwent surgery followed immediately by chemotherapy. The CK19^+ ^cell numbers were tested before surgery, 7 days after chemotherapy and 90 days after chemotherapy.(A and C) Patients with CK19 positive cells before surgery; (B and D) Patients without CK19 positive cells before surgery. Different symbols represent different breast cancer patients. The data were analyzed by the K Related Samples Test, **, p < 0.01 (A).

The dynamic expressions of CK19 in peripheral blood lymphocytes were observed in 15 patients with primary breast cancer during 3 month-chemotherapy after lumpectomy. The number of CK19^+ ^cells among 10^5 ^leukocytes of each patient was presented in Figure 6. For the 7 metastatic patients, there was significant difference in CK19 expression level before and after clinical treatment (p = 0.001). The CK19^+ ^cell numbers were obviously decreased after operation and chemotherapy, and there was almost none 3 months later (Figures 6A and 6C). For the 8 patients without CK19^+ ^cells before surgery, no significant difference was seen after clinical treatment (p = 1). The numbers of CK19^+ ^cells of 6 patients were always nearly zero during 3 month-chemotherapy, but increased in 2 patients after treatment (Figures 6B and 6D).

## Discussion

The dispersion of tumor cells is one of the primary causes of recrudescence at distant sites and of death from cancer. So the detection of occult metastatic cells is important to predict recurrence and improve survival. In this study, we applied flow cytometry to examine the expression of CK19 in the peripheral blood of breast cancer patients to monitor CTCs.

Immunocytochemistry gives morphological detail of tumor cells but is not sensitive and lack of methodological standardization [18]. Although RT-PCR is able to find 1 cancer cell among 10^6 ^irrelevant cells [19], it cannot exactly quantify the number of tumor cells according to mRNA levels. Furthermore, its utility was limited for its low specificity because of the false positive results which may be explained by the phenomenon of "illegitimate expression" [20,21].

In the present study, flow cytometry is utilized to examine the expression of CK19 to test CTCs in 48 breast cancer patients because most breast cancer cells but not blood cells express CK19. Although the sensitivity of our method is 1 cancer cell among 10^4 ^irrelevant cells, its specificity is very high. No CK19 expression was detected in healthy volunteers and patients with benign tumor. We consider high specificity is more important than high degree of sensitivity for clinical diagnoses because a wrong positive test will result in unnecessary treatments that may cause injury. Our data demonstrated that 86% of stage IV patients and 70% of stage III patients were detected CK19^+ ^cells in the peripheral blood, which were a little higher than that reported by Aerts J [22]; but the percentage of patients at stages I and II was lower. This may be explained by that the detection of protein level is more precise than RNA level because of the false positive result of RT-PCR also reported by others [23]. In our study the percentages of CK19^+ ^cells in the peripheral blood samples of patients were increased as the illness grew worse. This result was similar with that of Ivy Wong and his group that positive expression level of CK19 correlates strongly with disease stage in colorectal cancer [24]. Moreover, most patients positive for CK19 had a tumor size of more than 2 cm. It was also mentioned by Weihrauch that CK19 detection rate increased with tumor size [25]. However, Xenidis N and his colleagues found the presence of CK19 positive cells had nothing to do with clinicopathological prognostic factors [26].

After a follow-up period of three month-chemotherapy, the number of occult tumor cells in most metastatic patients was decreased rapidly, convincing the effect of adjuvant chemotherapy. In another hand, this can also be considered that most CTCs are apoptotic [27] so they vanished automatically. However, 2 patients with no metastasis before operation had CK19 positive cells after chemotherapy. It may be explained by that chemotherapy may evoke the exudation of proinflammatory cytokines which can regulate gene expression [28]. The tumor cells of one patient vanished after durative chemotherapy, but for the other patient they increased during this treatment. This phenomenon indicates that some tumor cells are sensitive to chemotherapy but others are resistant to it.

In conclusion, we have established a simple method for the test of CTCs in peripheral blood. Despite its sensitivity seems not as high as PCR, the specification and quantification accuracy is encouraging. Our technique can also be applied for bone marrow metastasis investigation. Many groups have reported the relationship of CK19^+ ^cells with reduced overall survival and risk of distant relapse. The detection of CTCs by flow cytometry in breast cancer may monitor disease progression and be helpful in the selection of patients who have the risk of relapse after adjuvant treatment.

## Conclusion

The presence of CTCs associates with clinicopathological factors such as tumor size and disease stage. The detection of CK19 in peripheral blood by flow cytometry is a specific and feasible method to monitor CTCs which relate to relapse and survival.

## Competing interests

The authors declare that they have no competing interests.

## Authors' contributions

LW performed the laboratory assays and drafted the manuscript. YW carried out the statistical analysis and revised the manuscript. MC conceived of the study and participated in its coordination. YL contributed to cell culture, image treatment and manuscript revision. XW participated in the use of LSCM. HW was the principal investigator of the study. All authors read and approved the final manuscript.
